# Longitudinal study of multidimensional factors influencing maternal and offspring health outcomes: a study protocol

**DOI:** 10.1186/s12884-023-05785-1

**Published:** 2023-06-22

**Authors:** Jianfei Chen, Ruixue Tian, Zhijie Zou, Jiaxin Wu, Jing Zhao, Yanlin Chen, Li Peng, Wenyi Lyu, Qiuxia Cheng, Zhongxiang Cai, Xiaoli Chen, Chunli Chen

**Affiliations:** 1 School of Nursing, Wuhan University, Located On No. 115, Donghu Road, , Wuhan, 430071 Hubei Province China; 2 Obstetrics and Gynecology Outpatient Clinic, Renmin Hospital of Wuhan University, Located On No.99 Zhang Zhidong Road, Wuchang District, Wuhan, 430060 Hubei China; 3 Department of Obstetrics and Gynecology, Renmin Hospital of Wuhan University, Located On No.99 Zhang Zhidong Road, Wuchang District, Wuhan, 430060 Hubei China; 4 Department of Nursing, Located On No.99 Zhang Zhidong Road, Wuchang District, Renmin Hospital of Wuhan University, Wuhan, 430060 Hubei China

**Keywords:** Longitudinal, Pregnancy, Mental health, Maternal health, Offspring

## Abstract

**Background:**

Reducing preventable adverse maternal and offspring outcomes is a global priority. The causes of adverse maternal and fetal outcomes are complex with multidimensional influencing factors. In addition, the Covid-19 epidemic has had a significant psychological and physical impact on people. China is now stepping into the post-epidemic era. We are curious about the psychological and physical situation of maternity in China at this stage. Therefore, we plan to initiate a prospective longitudinal study to investigate the multidimensional influences and mechanisms that affect maternal and offspring health.

**Method:**

We will recruit eligible pregnant women at Renmin Hospital of Hubei Province, China. The expected sample size is 1490. We will assess socio-demographics, Covid-19 related information, social capital, sleep, mental health and medical records, including clinical examination and biochemical tests. Eligible pregnant women will be enrolled in the study with less than 14 weeks of gestation. Participants will receive a total of nine follow-up visits between mid-pregnancy and one year postpartum. The offspring will be followed up at birth, 6 weeks, 3 months, 6 months and one year. In addition, a qualitative study will be conducted to understand the underlying causes that affect maternal and offspring health outcomes.

**Discussion:**

This is the first longitudinal study of maternity in Wuhan, Hubei Province which integrates physical, psychological and social capital dimensions. Wuhan is the first city to be affected by Covid-19 in China. As China moves into the post-epidemic era, this study will provide us with a better understanding of the long-term impact of the epidemic on maternal and offspring health outcomes. We will implement a range of rigorous measures to enhance participants’ retention rate and ensure the quality of data. The study will provide empirical results for maternal health in the post-epidemic era.

**Supplementary Information:**

The online version contains supplementary material available at 10.1186/s12884-023-05785-1.

## Background

The World Health Organization (WHO) has published a series of guidelines emphasizing the importance of perinatal health care for pregnant women and offspring [1, 2]. Along with China's continuous socio-economic development, the Chinese government and the public are increasingly focusing on maternal and child health outcomes. China's 13th Five-Year Plan [3] and the Health China Operation (2019–2030) [4] mentioned strengthening maternal and child health care and reproductive services. The Chinese government has placed considerable significance on perinatal health care. For example, at the recent 20th Congress of the Chinese government, establishment of a more comprehensive maternity support system was proposed [5].

Maternal and offspring health outcomes are affected by many risk factors, but the mechanisms are unclear. In 1977, Professor Engel developed the biopsychosocial model of medicine [6]. Since then, the etiology of health outcomes has been interpreted in a new approach. Without exception, previous studies have shown that maternal and offspring health is equally determined by biological, psychological and social factors. The first category is biological factors [7–9]. For example, Yasushi Tsujimoto et al. found that women who were obese and had gestational diabetes had a higher risk of preterm birth (PTB), low birth weight (LBW) and small for gestational age (SGA) [9]. The second category is psychological factors. In recent years, the study of gut microbiome has gained momentum. Previous studies have shown that the gut microbiome of young children with attention deficit hyperactivity disorder (ADHD) [10] and autism spectrum disorders (ASDs) [11] is different from that of the healthy children. Our team have also found that correlation between gut microbiome and temperament in 1–2 years old children [12]. We intend to further explore the extensive impact of gut microbiome and maternal and infant health outcomes. Perinatal depression, anxiety, fear of delivery, and stress can lead to spontaneous preterm delivery and low birth weight in pregnant women; and can cause cognitive-emotional-behavioral problems in the offspring [13]. The last category is social factors. This study attempts to use social capital to examine the impact of social factors on maternal and offspring health outcomes. Social capital refers to the associations between individuals or groups and the resource that people bring to their position in the social structure [14]. In recent years, social capital theory has been increasingly used as an important theory in health research [15], but with less application in the field of perinatal health in China. Hajar Pasha et al. have shown that pregnant women with low social capital report higher levels of stress [7]. Previous studies have shown numerous factors influencing maternal and offspring health outcomes. However, some of the studies are cross-sectional or retrospective, which can lead to inaccurate relationships between risk factors and the outcome.

Prospective longitudinal studies can provide evidence that suggests causality and information regarding the strength of the association between the risk factors and the outcome [16]. In the late twentieth century, large maternal longitudinal studies were established in Europe [17], the United States [18] and Australia [19]. These longitudinal studies have provided a wealth of research findings and evidence [17, 19]. In recent years, China has also launched a number of maternal and offspring longitudinal studies. The most representative of these are the China Birth Cohort Study (CBCS) [20] and the Chinese Pregnant Women Cohort Study (CWPCS) [21], which have established cohorts in the majority of Chinese provinces and provided rich data. In addition, there are several regional pregnancy longitudinal studies such as in Anhui [22] and Shanghai [23]. Therefore, it is imperative to launch a longitudinal study of maternal and offspring health outcomes in Wuhan that encompasses biological, psychological, and social capital dimensions.

Furthermore, in response to the appeal of the Department of Sexual and Reproductive Health and Research (SRH) at WHO to strengthen and expand existing pregnancy research platforms in the post-epidemic era [24], we initiated a prospective longitudinal study in Wuhan, which was the first city to fight the COVID-19 pandemic in China. We are interested in the impact of COVID-19 on maternal and offspring health outcomes, especially as China and the world move into the post-epidemic era. Our vision is to establish a prospective longitudinal study with sustainable follow-up; to provide rich and multidimensional data on maternal and offspring health outcomes; and to attempt to explain how women's multidimensional exposures during pregnancy affect maternal and offspring health outcomes.

### Aims

1. To draw the overall trajectory of maternal mental health conditions during the perinatal period, and to determine the potential phenotypes of the overall development trajectories of maternal mental health conditions. Subsequently, the relationship between mental health trajectories and maternal and offspring health outcomes will be further investigated.
2. To analyze the influence of social capital level on maternal and offspring health outcomes.
3. To analyze the impact of the biological factors of pregnant women on maternal and infant health outcomes. In addition, this study will explore the relationship between gut microbiome and maternal and infant health outcomes.
4. To integrate the effects of multidimensional influence factors including physical factors, psychological factors and social capital on maternal and offspring health outcomes. Then, to generate a prediction model for the risk of adverse maternal and offspring health outcomes.

**Fig. 1:**
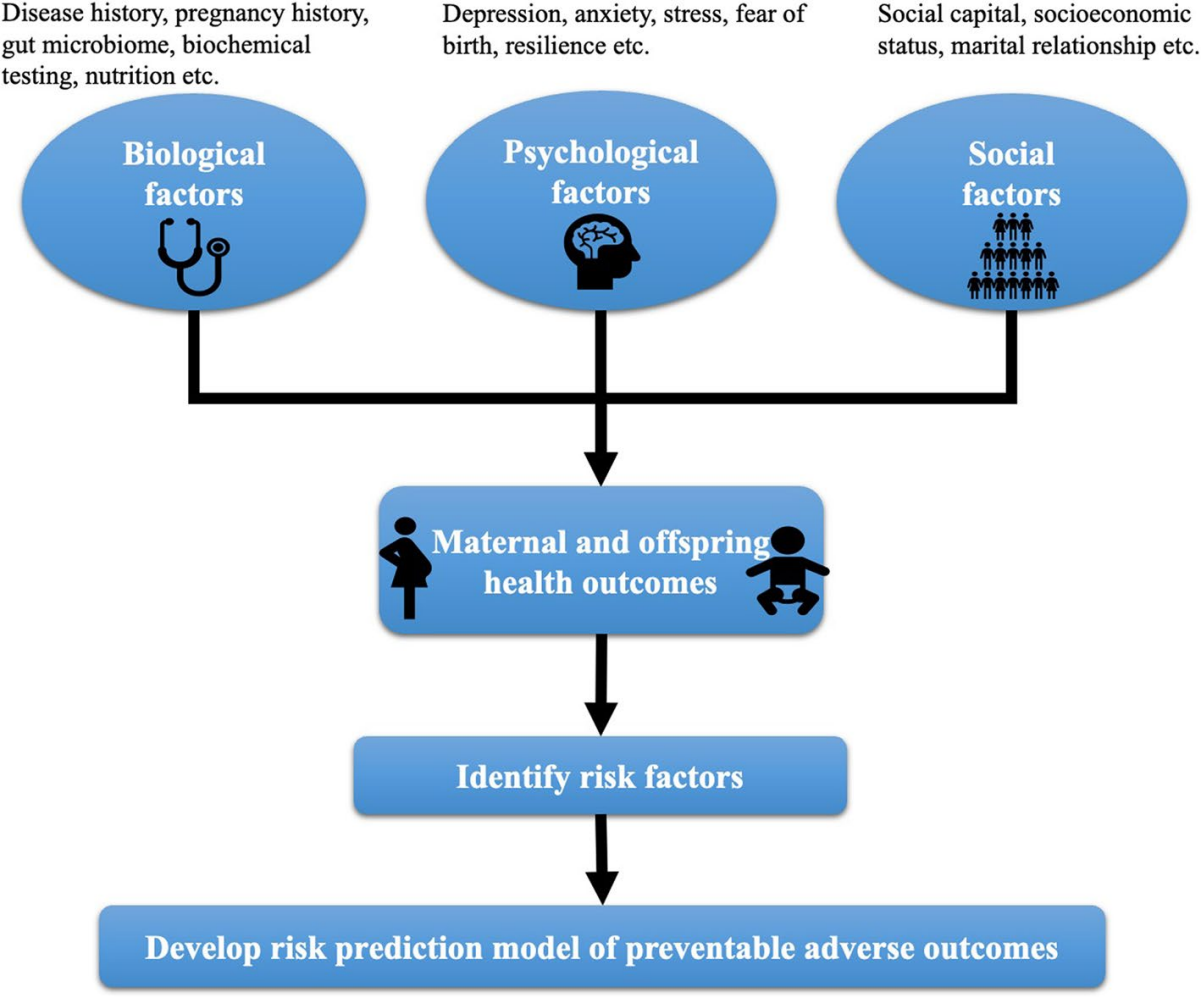
Conceptual framework

The conceptual framework of this study is shown in Fig. 1.

## Methods

### Study design

This study is a prospective longitudinal study. Eligible pregnant women will be recruited at the first trimester of gestation (gestational week < 14 weeks) from the obstetric clinics of Renmin Hospital of Hubei Province, and will be followed up until one year after delivery.

### Study sites

Our study will be conducted at the Renmin Hospital of Hubei Province, which is an affiliated hospital of Wuhan University, located in Wuhan, Hubei Province, China. We will conduct the study in both the obstetrics outpatient and inpatient obstetrics units of this hospital. Maternal recruitment and follow-up will be conducted in the outpatient clinic or via WeChat.

### Participant eligibility

All eligible pregnant women who reside in Wuhan will be considered as the study population of this study. All pregnant women who register and plan to receive regular perinatal medicine examinations at the hospital predetermined by researchers will be recruited as study participants.

#### Inclusion criteria

Eligible pregnant women must meet the following inclusion criteria:

1. Aged 18—50 years;
2. Less than 14 weeks of gestation;
3. Planning to receive perinatal medicine examinations and delivery at predetermined hospital;
4. Able to read and complete questionnaires in Chinese;
5. Having access to smart phone and internet;

#### Exclusion criteria

Pregnant women who plan to leave the study area (Wuhan) for delivery or postpartum recuperation.

### Sample size

We will use the following formula for the sample size calculation [25].

$$n={\left(\frac{{U}_{\alpha /2}}{\delta }\right)}^{2}\pi \left(1-\pi \right)$$

Through reviewing the literature, a meta-analysis by Xiang Lin et al. found that the rate of perinatal depression in Chinese pregnant women was 17.4% [26]. Significance level ⍺ = 0.05 and the tolerance error δ = 0.04 was set to perform the calculation. The estimated sample size needed is 345, and considering a 15% dropout rate at each follow-up (9 follow-ups in total), at least 1490 participants need to be included in the study at baseline.

### Study procedure

#### Recruitment

The researchers will screen and recruit participants from obstetric clinics in Renmin Hospital of Hubei Province. The recruitment has started in August 2022 and will continue until December 2023.In the outpatient department, pregnant women will be invited to participate in during the first prenatal examination (during 8–12 weeks of pregnancy). After that we will introduce the purpose and content of this study to each pregnant woman who are eligible to participate. Pregnant women who participate in will be asked to sign informed consent.

Then the researcher will perform a baseline assessment. Questionnaires are used to collect demographic information, Covid-19 related information, social capital, psychological condition, physical condition, sleep and nutrition. In addition, we will also collect feces from a part of pregnant women to perform gut microbiota testing. At the end, the patient's medical records (e.g., family history, disease history, pregnancy and childbirth history, etc.) will be extracted.

#### Follow-up procedure

After enrollment, we will follow up at 16–20 weeks, 21–24 weeks, 28–36 weeks and 37–40 weeks of gestation in prenatal period. The pregnancy follow-up visit will consist of different scales. We will contact the pregnant woman by telephone or WeChat to obtain a time for them to come for their maternity check-up. Pregnant women will be followed up when they come to the obstetrics clinic for their maternity check-up. If a pregnant woman cannot be followed up in hospital due to particular circumstances, we will do this via internet. We will use "Wenjuanxing", an online information collection system, to complete the online information gathering.

After delivery, we will follow up at 7 days, six weeks, three months, six months and one year postpartum. In addition to the mother's questionnaires and medical records (pregnancy complications, obstetric complications etc.), we also collect offspring medical records (gender, length, weight, head circumference, disease history etc.) and a mother-reported offspring scale (CSBS-DP) to reflect the offspring socialization development.

#### Medical records

The medical records are reported by specialist obstetricians. Investigators will collect records from the Hospital Information System (HIS). At baseline, the pregnant woman's family history, disease history, and pregnancy history will be collected. After delivery, maternal pregnancy complications, obstetric complications, biochemical test results; neonatal complications, weight, length, and head circumference of the newborn will be collected. Offspring growth and developmental indicators and history of disease during the first year postpartum will be collected at one year.

**Fig. 2:**
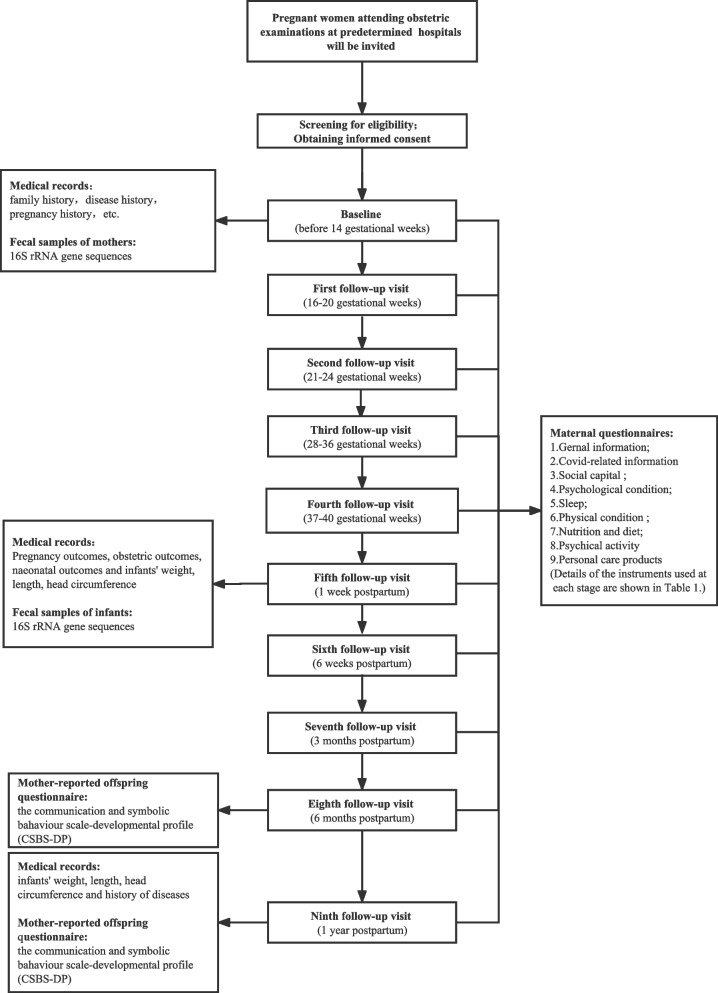
Study procedure flowchart

The flow chart (Fig. 2) shows the study procedure from participant recruitment to the end of the study.

### Study instruments

We will use several instruments to assess the intended measures as described below. The various tools used for each follow-up period are shown in Table 1.

1. General information
  *Self-designed maternal general information questionnaire*: pregnant women’s socio-demographic characteristics, medical history and pregnancy-related characteristics.
2. Covid-related information
  *Self-designed Covid-related information questionnaire*: history of Covid-19 infection in pregnant women and family members, level of anxiety about the impact of the Covid-19 infection on offspring.
3. Psychological conditions
  3.1 Edinburgh Postnatal Depression Scale (EPDS) [27]: Validated Chinese version of the Edinburgh Postnatal Depression Scale [28].
  3.2 Pregnancy-related Anxiety Questionnaire (PRAQ): Developed and validated by Chinese scholar Limin Xiao et al. [29].
  3.3 Generalized Anxiety Disorder 2 items (GAD-2) [30]: Validated Chinese version of GAD-7 was developed by Xiaoyan He et al. [31]. GAD-2 was chosen from the first two items of GAD-7. The reliability and validity of the GAD-2 have been confirmed by Kroenke et al. [30].
  3.4 Fear of Birth Scale (FOBS) [32]: Chinese version were translated by Lixia Qu et al. [33].
  3.5 Connor-Davidson Resilience Scale 10items (CD-RISC-10) [34]: Chinese version of CD-RISC-10 was translated and validated by Zengjie Ye et al. [35].
  3.6 Perceived Stress Scale (PSS-10) [36]: Chinese Perceived Stress Scale were translated, adapted and validated by Yanzhong Yang et al. [37].
4. Social capital
  *Personal Social Capital Scale 16 (PSCS-16)*: Developed and validated by Chinese scholar Peigang Wang et al. [38].
5. Sleep
  *Insomnia Severity Index (ISI)* [39]: Validated Chinese version were translated by Kai-Fai Chung et al. [40].
6. Nutrition and diet
  *Food Frequency Questionnaire (FFQ)*: Validated and adapted version in Hubei, China [41].
7. Oral health
  *Oral Health Impact Profile (OHIP-14)* [42]: Chinese version of OHIP-14 was translated and validated by Weini Xin et al. [43].
8. Urinary incontinence
  *International Consultation on Incontinence Questionnaire Short Form (ICIQ-SF)*: Chinese version of ICIQ-SF was translated and validated by Zebo Chen et al. [44].
9. Physical activity
  *International Physical Activity Questionnaire Short Form (IPAQ-SF)*: Chinese version of IPAQ-SF were translated and validated by Ningning Qu et al. [45].
10. Personal care product
  *Personal care products questionnaire (PCPQ)*: Referring to the study by Zorimar Rivera-Núñez et al. to develop self-design PCPQ in Chinese [46]. PCPQ included whether or not pregnant women used perfumes, soaps, lotions, cosmetics, nail polish, shampoos and conditioners, hairsprays, mouthwashes, hair dyes.
11. Offspring assessment
  *The Communication and Symbolic Behavior Scale-Developmental Profile (CSBS-DP)* [47]: Chinese version of CSBS-DP was translated and validated by Chusui Lin et al. [48].

**Table 1:** Study instruments

Study instruments	Timepoint									
	Pregnancy Period					Postpartum Period				
	Enrollment	16–20 weeks	21–24 weeks	28–36 weeks	37–40 weeks	1 week	6 weeks	3 months	6 months	1 year
*Demographic information*	✕									
*Covid-related information*	✕					✕				
*Psychological conditon*										
EPDS	✕	✕	✕	✕	✕	✕	✕	✕	✕	✕
PRAQ	✕		✕		✕					
FOBS	✕	✕	✕	✕	✕					
GAD-2		✕	✕	✕	✕	✕	✕	✕	✕	✕
CD-RISC-10		✕		✕	✕		✕		✕	
PSS					✕		✕		✕	
*Social capital*										
PSCS-16	✕		✕		✕	✕		✕		✕
*Health status*										
OHIP-14			✕							
ICIQ-SF				✕	✕	✕		✕		✕
ISI	✕		✕		✕	✕		✕		✕
*Life style*										
IPAQ-SF	✕	✕		✕		✕		✕		✕
PCPQ		✕								
FFQ	✕									
*Infant assessment*										
CSBS-DP									✕	✕

*PSCS-16* Personal social capital scale-16, *ISI* Insomnia severity index, *EPDS* Edinburgh postnatal depression scale, *PRAQ* Pregnancy-related anxiety questionnaire, *FOBS* Fear of birth scale, *GAD-2* Generalized anxiety disorder 2 items, *CD-RISC-10* Connor-davidson resilience scale 10items, *PSS* Perceived stress scale 10 items, *ICIQ-SF* International consultation on incontinence questionnaire short form, *OHIP-14* Oral health impact profile 14 items, *PCPQ* Personal care products questionnaire, *FFQ*: Food frequency questionnaire, *CSBS-DP* The communication and symbolic behavior scale-developmental profile

### Laboratory testing

Routine maternal blood, urine and stool screening procedures will be performed in the laboratory departments of Renmin Hospital of Hubei Province. In addition, we will conduct studies related to maternal and offspring feces for gut microbiome. The fecal samples will be transported in a cooler (+ 4℃, 1.5 h on average) to Wuhan University Maternal and Child Health Research Centre and then stored in a -80°C refrigerator. The universal primers 338F (5’-ACTCCTACGGGAGGCAGCA-3’) and 806R (5’-GGACTACHVGGGGTWTCTAAT-3’) were used to amplify the 16 s rRNA gene (V3-V4) fragments of bacteria from fecal samples. Wuhan University Maternal and Child Health Research Centre has extensive experience in the field of Gut Microbiota research in recent years [12, 49–52].

### Qualitative exploration

A qualitative study can help us to understand more deeply the hidden meaning behind the data. The subjects for the qualitative study will be generated among the pregnant women participating in our study. The semi-structured interview outline will be developed by a sociologist in the team and the interviews will be conducted by trained interviewers. The topics we propose to undertake a qualitative exploration of the role that social capital plays in maternal health, the psychological impact of the Covid-19 epidemic on maternity, and the parenting experiences of women with perinatal depression etc. Our interviews will be conducted according to the COREQ checklist [53], with strict adherence to the quality of the qualitative study. We will analyze interview data in depth, refine themes, and add explanations for incompleteness of quantitative research. This qualitative study will help us further understand how multidimensional factors affect maternal and fetal health conditions.

### Statistical analysis

The baseline characters of continuous variables will be described with mean and standard deviation (SD), while the categorical variables will be described by frequency and percentage (%). We will conducte the student t test or one-way analysis of variance (ANOVA) for continuous variables with normal distribution to examine the difference among two or three groups. And the Mann–Whitney U test will be performed to determine the statistical significance for continuous variables with skewed distribution. The chi-square test (χ2) and Fisher-Freeman-Halton exact test will be conducted for the difference of categorical variables. For the significant outcome variables, we will conducted a multi-variable logistic regression model with discrepant characters adjusted to explore the association between pregnancy and neonatal outcomes. And the Odds ratios (ORs) along with its 95% confidence intervals (CIs) will be calculated to quantify the association. We will use the Directed Acyclic Graph (DAGs) method to identify confounding, mediating, or impulsive variables and use this to control confounding and ensure equation stability. And a two-sided p-value less than 0.05 will be defined with statistical significance. We will use the latent class growth model (LCGM) when plotting and categorizing the trajectory of perinatal mental health status. Data from women who are lost to follow-up will also be included in the analysis to reduce bias. For missing values, we will use deletion, multiple interpolation or no processing, as appropriate. We will also use sensitivity analysis to ensure the accuracy of the results. All the above statistical analysis will be performed using SPSS 26.0, Mplus 8.0 and R 4.0.2.

For the qualitative study we will use the Colaizzi phenomenological seven-step analysis: (1) acquaint with all the interview data and transcribe the audio recordings (within 12 h after the interview); (2) analyze the interview notes and extract meaningful statements; (3) summarize and refine the statements and code them; (4) look for concepts with similarities and form themes from the coded views; (5) describe the themes in combination with the study phenomenon. (6) summarizing similar views; (7) verifying the themes at the study population we had interviewed. The data analysis will be conducted by two researchers, and in case of disagreement, the group members then participated in the discussion to develop the final themes.

### Quality control

When collecting data in the obstetric clinic, we ensure the accuracy of the data by using standardized scales and training the data collectors. At each study site, we arranged for a core investigator and two data collectors. The core investigator is assigned who is responsible for resolving any problems encountered by the data collectors during the collection process in the first instance. After the participants had completed the scales, the data collectors should timely check for any missing items. The data will be entered into the database every evening and checked again as they are input. We use EpiData 3.1 to build our database, which is a reliable data entry and management software. After the input is completed, the accuracy of the data in the database is randomly checked daily.

### Ethical considerations

This study is a prospective longitudinal study in which there are several ethical considerations. First, a detailed description of the study and process will be given to all pregnant women participating in this study, and a written consent will be obtained. Second, our study is conducted in parallel with the regular maternal healthcare program. This is an observational study and no intervention is imposed. Last, any abnormal indicators found during the study will be reported to the corresponding responsible doctor or nurse of the pregnant woman. This study has been approved by the Ethics Committee of Wuhan University School of Medicine. The ethical approval number is WHU2021-YF001.

### Patient and public involvement and engagement(PPIE)

During the study, especially when designing the questionnaire, we consulted pregnant women to avoid situations where some items were controversial or difficult to understand. We also conducted a pre-survey and asked pregnant women again if there were any improvements to the questionnaire.

We conducted two seminars with experts in the field of public health. The public health experts we invited had extensive experience in social capital and longitudinal studies. We also held two seminars with hospital administrators, doctors, and nurses. We discussed in detail with doctors and nurses the specific format and details of the study we were conducting. We received full understanding and support from the hospital administrators. The full cooperation with the hospital contributed to the successful implementation of our study.

This study was funded by the National Social Science Foundation of China. Therefore, we could have research funding and gain the trust and cooperation from the administration, medical institutions and the public.

## Discussion

This study was pre-investigated and confirmed to be feasible. In July 2022, we conducted a pre-survey of the questionnaires and a pilot test. We made modifications to several problems identified in the questionnaire and optimized the process of data collection, entry and storage. We trained our research team, including doctors, nurses and midwives, and enhanced their understanding of the study purpose and implementation procedures. In addition, we produced promotional posters and highlighted the benefits of pregnant women's participation in our research: identifying potential effects of multiple exposure factors on pregnant women and fetus. This method could allow more pregnant women to participate in our studies. Our pilot study confirmed the actionability of our project.

 Longitudinal data can help us better understand how multiple exposure factors (e.g., social capital, mental health, etc.) impact on pregnancy and offspring outcomes. Large sample sizes and richly researched maternal longitudinal studies have been conducted in countries such as the USA [18], UK [17], Australia [19], Sri Lanka [54] and South Korea [55] etc. To the best of our knowledge, the largest study in China is the China Birth Cohort Study (CBCS) [20]. CBCS was conducted in 17 provinces in China, with 38 hospitals participating, and included over 120,000 pregnant women, but they were not included in the assessment of mental health. The second largest Chinese maternal cohort is the Chinese Pregnant Women Cohort Study (CPWCS) [21], which was conducted in 24 hospitals across China in 2017. The rest of the longitudinal studies are regional such as the China-Anhui birth cohort (C-ABC) [22] and the Shanghai birth cohort [23].

Comparing with other longitudinal studies in China, this study has the following advantages. First, we will follow from the first trimester to one year postpartum, with a total of ten sessions and measurement of multiple exposure factors. Our results will provide a more detailed understanding of maternal health at all stages of pregnancy, in order to provide the strongest evidence of the multidimensional health of the mother and offspring. Second, we correlate social capital with maternal and offspring health outcomes. We attempt to explain the complex causes of health problems from a sociological perspective. This will help us to reduce disparities in society in order to build more equal and well-developed maternal health systems. Third, we plan to analyze the role that gut microbiome plays in maternal and offspring health outcomes. We are particularly interested in the impact of gut microbiome species and abundance on the psychosocial development of the offspring. This will provide a broader perspective and approach to optimize the development of the offspring. Finally, this study is a prospective longitudinal study of maternal and offspring health outcomes that encompasses biological, psychological, and social capital dimensions. We will eventually develop a predictive model for adverse maternal mental health outcomes based on the study results and validate it in the population. The model will further reduce the incidence of adverse maternal events in our country and indeed worldwide.

Moreover, considering the abundant data generated by our multidimensional maternal longitudinal study, our research will explore various areas of maternal and child health in greater depth. Currently, we have plans to conduct studies based on sub-samples in diseases such as Novel coronavirus pneumonia, heart disease, hepatitis B, thyroid disease, diabetes and hypertension. We are confident that the wealth of data generated will contribute to a considerable amount of worthwhile research in the future. The findings will better benefit pregnant women and future generations in China and around the world.

## Supplementary Information

**Additional file 1. **Reporting checklist for cohort study.

## Data Availability

Data sharing is not applicable to this article as no datasets were generated or analysed during the current study.
